# Chemokines in Chronic Liver Allograft Dysfunction Pathogenesis and Potential Therapeutic Targets

**DOI:** 10.1155/2013/325318

**Published:** 2013-12-08

**Authors:** Bin Liu, Jing Li, Lu-Nan Yan

**Affiliations:** ^1^Department of General Surgery, General Hospital of Tianjin Medical University, Tianjin 300052, China; ^2^Department of Anesthesiology, General Hospital of Tianjin Medical University, Tianjin 300052, China; ^3^Department of General Surgery, West China Hospital, Sichuan University, Chengdu, Sichuan 610041, China

## Abstract

Despite advances in immunosuppressive drugs, long-term success of liver transplantation is still limited by the development of chronic liver allograft dysfunction. Although the exact pathogenesis of chronic liver allograft dysfunction remains to be established, there is strong evidence that chemokines are involved in organ damage induced by inflammatory and immune responses after liver surgery. Chemokines are a group of low-molecular-weight molecules whose function includes angiogenesis, haematopoiesis, mitogenesis, organ fibrogenesis, tumour growth and metastasis, and participating in the development of the immune system and in inflammatory and immune responses. The purpose of this review is to collect all the research that has been done so far concerning chemokines and the pathogenesis of chronic liver allograft dysfunction and helpfully, to pave the way for designing therapeutic strategies and pharmaceutical agents to ameliorate chronic allograft dysfunction after liver transplantation.

## 1. Introduction 

Chronic liver allograft dysfunction is a leading cause of patient morbidity and late allograft loss after liver transplantation. The loss of approximately 2000 liver grafts each year results in chronic allograft dysfunction [1]. Liver allograft biopsy in patients who survive longer than 5 years shows that 37% of recipients present with chronic liver allograft dysfunction [2].

The pathological hallmarks of end stage chronic liver allograft dysfunction include hepatocyte necrosis, hepatic arterial proliferative occlusive disease, bile duct disappearance, and eventually liver fibrosis [3]. That pathological changes usually precede functional deterioration in cases of chronic liver allograft dysfunction is characterized [3]. Treatment options in patients with advanced chronic liver allograft dysfunction are limited because of the diffuse nature of the disease. The currently available drug treatments are ineffective. Additionally, retransplantation has limited applicability and success because of donor availability. Hence, chronic liver allograft dysfunction still is a common and frequently fatal, yet poorly treatable, complication of liver transplantation.

Although the pathogenesis of chronic liver allograft dysfunction is not completely defined, it is believed that the histopathologic changes in this patient population can be attributed to early allograft dysfunction [4], acute or chronic rejection [5, 6], de novo or recurrent autoimmune disease [7], de novo or recurrent viral hepatitis [3], drugs toxicity [8, 9], late effects of ischemia/reperfusion (I/R) injury [10] or ischemic-type biliary lesions [11, 12], and other recurrent diseases [13]. Causes of chronic liver allograft dysfunction are variable and are shown in Table 1. The molecular mechanisms of chronic liver allograft dysfunction are still unclear. Several reports have shown that chronic liver allograft dysfunction is caused by repeated episodes of chemotactic mediated injury to the liver graft [14, 15]. And these forms of injury are inflicted on the allograft throughout all stages of transplantation [16].

Chemokines are a group of low-molecular-weight (8 to 14 kDa) [17] cytokines of which their common properties are to induce inflammatory cells migration and regulate inflammatory responses. However, recent studies show that chemokines impinge on many facets of biology including angiogenesis, haematopoiesis, mitogenesis, tumour growth and metastasis [14, 18], and participating in the development of the immune system and in innate and acquired immune responses [19, 20]. Dysregulated expression of chemokines and their receptors is involved in the pathogenesis of many human diseases including chronic inflammatory diseases, autoimmune diseases, immunodeficiency, and cancer. Furthermore, chemokines are essential mediators for attracting immune cells and for activating nonparenchymal liver cells [21]. And there is also emerging evidence that these chemokines and their receptors are linked with chronic liver allograft dysfunction development in animal studies [16].

For their involvement in a number of pathological processes, chemokines and their receptors represent important pharmaceutical targets for many diseases [65]. In addition, genetically recombinated/engineered small-molecule chemokine or chemokine inhibitors are emerging in reports both in the literature and at international conferences. In this review we will outline the recent progress in chemokines research with regard to the pathogenesis and development of chronic allograft dysfunction after liver transplantation. Each class of chemokines is discussed separately in this paper.

## 2. Chemokine Superfamily

Since the first member of chemokines/cytokines, platelet factor 4 (PF-4/CXCL4), being discovered in 1955 [66], the family members of chemokines are more than 50 now. Because these molecules are closely related in structure and function, enormous chemokines and chemokine receptors were newly discovered in recent years. According to the presence of a conserved cysteine residue at the NH_2_ terminus [67], the chemokine superfamily is divided into four subfamilies: C, CC, CXC, and CX_3_C. The first and third cysteines are missing in the C subfamily, while these two cysteines are adjacent in CC chemokines. In the CXC subfamily, one amino acid separates the first two cysteines, while in CX_3_C chemokines, three amino acids between the two cysteines. Based on the presence or absence of a three-amino-acid sequence ELR, comprising glutamine (E), leucine (L) and arginine (R), adjacent to the CXC motif near the NH_2_ terminal, the CXC family is further subdivided into ELR-positive and ELR-negative CXC chemokines. A possible new type of CX chemokine, which lacks one of the two N-terminus cysteines but retains the third and fourth ones, has recently been reported in Zebrafish [68], but there is no evidence that this kind of chemokine exists in mammals.

According to the subfamily of their major ligands, the chemokine receptors are also classified into four subfamilies [17]. They are generally a kind of 7-transmembrane-spanning G proteins, which are composed of *α*, *β*, and *γ* subunits. The chemokine can activate downstream signal transduction events following the interaction with its receptor (leading to the exchanging of GTP for GDP between different subunits of the receptor and dissociation of the *α* subunit from the *β* and *γ* subunit) [69]. The chemokines tend to have multiple chemokine receptors and some receptors also have large numbers of chemokine ligands [70]. The subfamily members of chemokines involved in the pathogenesis of liver disease are summarized in Table 2.

## 3. The CXC Chemokine Family 

### 3.1. The CXC Chemokines and Early Liver Allograft Dysfunction

ELR containing (ELR^+^) CXC chemokines, known as chemoattractants for neutrophils [71, 72], are distinct from other CXC chemotactic cytokines by the presence of the sequence glutamic acid-leucine-arginine (ELR) near the amino terminal. And the majority of ELR^+^ CXC chemokines bind to CXC chemokine receptor 2 (CXCR2) [73]. Significant time-dependent upregulation of CXCL1 was identified in the brain-dead donor livers [74]. However, these local inflammatory responses can lead to primary allograft dysfunction [75] after organ transplant from a brain-dead donor. CXCL2, known as macrophage inflammatory protein (MIP)-2, has been identified to stimulate tumor cell migration *in vitro* and angiogenesis and tumor growth *in vivo *[76]. Nevertheless, elevated levels of CXCL2 in graft livers have also been associated with ischemia/reperfusion injury after liver transplantation [77]. CXCL2 and its receptors are important mediators involved in neutrophil-dependent hepatic injury induced by ischemia and reperfusion [78]. Additionally, the pulmonary injury after liver transplantation was identified in mice and humans. Further studies have suggested that cold ischemia time prolongation upregulates pulmonary CXCL2 expression via hepatic-derived tumor necrosis factor-*α* (TNF-*α*) and promotes neutrophils accumulation resulting in increased pulmonary injury after liver transplantation [79]. CXCL8 promotes rapid liver regeneration after drug-induced acute injury and may have tremendous clinical potential in reducing the need for liver transplantation and the mortality associated with acetaminophen-induced fulminant liver failure [80]. Collectively, these studies suggest that ELR^+^ CXC chemokines and their receptors, axis may play an important role in neutrophil recruitment and mediate early allograft injury, which is a known risk factor for the pathogenesis and development of chronic liver allograft dysfunction.

ELR^−^ CXC chemokines include CXCL9, CXCL10, CXCL11, CXCL12, CXCL13, and CXCL14. Their receptors, including CXCR3, CXCR4, CXCR5, and CXCR7, are expressed predominantly on Th1 cells, Th2 cells, Th17 cells, Treg cells, memory T cells, some B cells, and natural killer cells [81]. The serum levels of CXCL9 and CXCL10 measured by Luminex multiplex assays increased in recipients with early allograft dysfunction after liver transplantation and correlated with T-cell recruitment [4]. Increased expression of CXCR3, CXCR4, and CCR5 has been shown on circulating and graft-infiltrating lymphocytes after liver transplantation [82]. These studies suggest that ELR^−^ CXC chemokines participate in lymphocyte recruitment in virtually all stages after transplantation and are involved in the retention of alloactivated lymphocytes at sites of graft damage, correlating with the pathogenesis early liver allograft dysfunction.

### 3.2. The CXC Chemokines and Acute Rejection after Liver Transplantation

Several experimental and clinical studies have implicated the ELR^−^ CXC chemokines/CXCR3 axis in reference to acute cellular rejection. Interestingly, full MHC-mismatched donor hearts had prolonged survival in CXCR3^−/−^ mice [83]. The similar results have been yielded when blocking antibodies to CXCR3 was used for heart allograft transplantation [84].

*In vivo *treatment with interferon-**γ** (IFN-*γ*) upregulates both hepatocyte and nonparenchymal cell (i.e., monocytes/macrophages, neutrophils, and other inflammatory cells) expression of CXCL9 [33, 85]. CXCL9 is strongly expressed on vascular and sinusoidal endothelium in rejecting hepatic allografts [82]. The interaction between CXCL9 and its receptor CXCR3 is important in recruiting lymphocytes to sites of inflammation within liver tissue [14]. The significant increasing expression of CXCL10, and CXCL11 and their receptor CXCR3 [86], together with the increase of B lymphocytes and plasma cells in liver biopsy specimens from patients with acute allograft rejection, indicates that the migration of B lymphocytes and plasma cellspromoted by the expression of chemokines/receptors, plays a key role in acute liver rejection [87]. Additionally, the chemokine CXCL11/CXCR3 axis has an important role in the homing of CD4(+) T cells [88] and NK cells [86] in acute rejection models of solid organ transplantation. However, when compared with the biological responses induced by CXCL9 or CXCL10, CXCL11 is of higher potency and efficacy in activated T cells and cells transfected with CXCR3 [14].

Collectively, the expression of CXCL9 and of other CXCR3 ligands (i.e., CXCL10 and CXCL11) is induced in rejecting hepatic allografts [82], with the increased expression of CXCR3 [89] on circulating and hepatic lymphocytes, suggesting that these chemokines may be therapeutic targets for acute liver allograft rejection.

### 3.3. The CXC Chemokines and Hepatic Ischemia Reperfusion (I/R) Injury 

Hepatic ischemia reperfusion (I/R) injury is an important clinical problem after liver resection or transplantation [90]. It can be categorized into warm I/R and cold storage reperfusion injury [91]. Furthermore, hepatic warm I/R injury can be subdivided into two distinct phases [92]. The initial phase (<2 h after reperfusion) is characterized by Kupffer cells (KC) mediated responses and oxidant stress, which results in the release of TNF-*α* [93]; the late phase of injury (from 6 to 48 h after reperfusion) is characterized by neutrophil accumulation and CXC chemokine production, which results in hepatocellular injury [94, 95].

**Figure 1:**
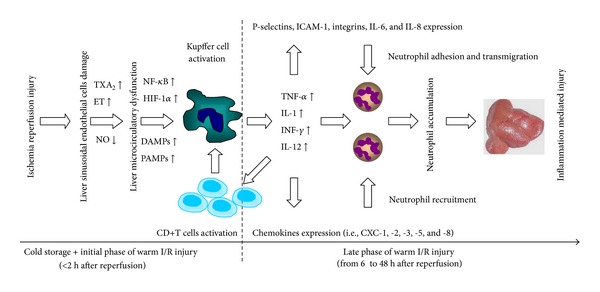
The inflammatory pathways of hepatic ischemia/reperfusion (I/R) injury. Liver sinusoidal endothelial cells (LSEC) damage, which occurs during cold preservation, represents the initial factor leading to liver I/R injury. Kupffer cell (KC) and LSEC edema, together with the imbalance between nitric oxide (NO) (↓) and thromboxane A2 (TXA2) (↑) and endothelin (ET) (↑), contributes to liver microcirculatory dysfunction. KC activation is promoted by damage-associated molecular patterns (DAMPs) (↑) and pathogen-associated molecular patterns (PAMPs) (↑) produced by neighbouring hepatic cells. Then activated KCs increase their release of ROS and proinflammatory cytokines including tumour necrosis factor-a (TNF-a), interleukin-1 (IL-1), interferon- (INF), interleukin-12 (IL-12), which induces the expression of P-selectin, intracellular adhesion molecule-1 (ICAM-1), integrins, IL-6, IL-8 in LSEC and the release of chemokines (i.e., CXC-1,-2,-3,-5, and -8). Additionally, IL-1 and TNF-a recruit and activate CD4+ T-lymphocytes, which amplify KC activation and promote neutrophil recruitment and adherence into the liver sinusoids and finally execute liver inflammation and injury.

Specifically, the last studies have suggested that liver sinusoidal endothelial cells (LSEC) damage, which occurs during cold preservation, represents the initial factor leading to liver I/R injury [90]. KC and LSEC edema, together with the imbalance between low nitric oxide (NO) bioavailability and exacerbated thromboxane A2 (TXA2) and endothelin (ET) production, contributes to liver microcirculatory dysfunction. KC activation is promoted by increased production of damage-associated molecular patterns (DAMPs) and pathogen-associated molecular patterns (PAMPs) by neighbouring hepatic cells [91]. Then activated KC significantly increase their release of ROS and proinflammatory cytokines including TNF-*α*, interleukin-1 (IL-1), IFN-*γ*, and interleukin-12 (IL-12) [92], which induces the expression of P-selectin, intracellular adhesion molecule-1 (ICAM-1), integrins, IL-6, IL-8 in LSEC, and overexpression of ELR containing CXC chemokines (i.e., CXC-1,-2,-3,-5, and -8) with their receptors CXCR1 and CXCR2 being expressed on neutrophils, SECs, and hepatocytes [96]. Additionally, IL-1 and TNF-*α* recruit and activate CD4+T-lymphocytes, which amplify KC activation and promote neutrophil recruitment and adherence into the liver sinusoids [97]. The inflammatory pathways of hepatic ischemia/reperfusion (I/R) injury are shown in Figure 1.

However, CXC chemokines regulate both the injury and recovery from I/R after liver surgery [7]. Ren and his colleagues have firstly reported that ELR^+^ CXC chemokines were shown to induce proliferation in hepatocytes in animal models [98]. These findings have also been identified in liver regeneration following 70% partial hepatectomy. When ELR^+^ chemokines were neutralized using antibodies, liver regeneration was impaired in the mass of remnant liver; conversely, hepatocyte proliferation and liver regeneration were accelerated by treatment of mice with CXCL2 after partial hepatectomy [98]. Additionally, pharmacological antagonism or genetic deletion of CXCR2 after hepatic I/R resulted in augmented hepatocyte proliferation and accelerated recovery from injury [99]. The chemokine receptor CXCR1 shares ligands with CXCR2. However, hepatocyte proliferation was decreased in CXCR1^−/−^ mice *in vivo* [100]. This study suggested that CXCR1 appears to facilitate repair and regenerative responses after I/R injury. CXC chemokines and their receptors have significant impact on potential therapeutic modulation of hepatic I/R injury.

## 4. The CC Chemokine Family

### 4.1. The CC Chemokines and Early Liver Allograft Dysfunction

Early allograft dysfunction (EAD) among liver transplant recipients is characterized by early high transaminases, persistent cholestasis, and prolonged coagulopathy [101, 102]. EAD occurring in the first week after liver transplantation is associated with increased graft failure and mortality and is often thought to be secondary to ischemia/reperfusion (I/R) injury [4]. Several perioperative factors, such as hypotension, reperfusion, donor brain death, and cold storage, contribute to the pathogenesis and development of EAD. Secondary to oxidative stress, cell death, and the release of inflammatory mediators in I/R injury, reactive oxygen intermediates are generated and the CC chemokines are released [103].

Twenty-eight CC chemokines have been characterized as up-to-date. And these CC chemokines were centrally involved in the activation or recruitment of T cells, NK cells, or B cells, depending on the biological context [104]. The expression of many CC chemokines in Kupffer cells is regulated by NF-*κ*B pathway activation [105]. Additionally, many CC chemokines are recently identified to play important roles in the pathogenesis of liver diseases.

Serum levels of peripheral blood CC chemokines, such as CCL2, CCL3, CCL4, and CCL5, pre- and postoperatively in patients with or without EAD were measured by Luminex multiplex assays [4]. Then the correlations were found between preoperative and postoperative expression of several chemokines and the development of EAD following liver transplantation. Although these serum proteins were showing significant change associated with EAD, it is not clear whether they represent the actual cause or effect of organ dysfunction or part of a new pathogenic process altogether. Further studies should be required to validate that these serum levels of peripheral blood CC chemokines are indeed part of a pathophysiologic mechanism with EAD.

### 4.2. The CC Chemokines and Acute Rejection after Liver Transplantation

Acute liver allograft rejection is characterized by a mixed portal tract infiltrate that contains mononuclear cells in human or rat liver transplant allografts (DA→Lewis orthotopic liver transplantation model). The accumulation of activated lymphocytes into the allograft plays an important role in the pathogenesis of tissue injury. Chemokines recruit the lymphocytes from the circulation and promote the migration, positioning, and retention of effector cells in the graft [82]. These chemotactic factors are expressed and secreted by a wide variety of cell types including lymphocytes [106] and endothelial components of rejecting allografts [107] in response to activation [108]. Several studies have shown that CCL2 (monocyte chemoattractant protein-1), CCL3 (macrophage inflammatory protein-1*α*), CCL3L1 [109], and CCL5 (RANTES) are upregulated in rejecting liver allografts [110]. Within the liver graft, chemokine-producing endothelial cells (CCL3, CCL4, and CCL5) and biliary epithelial cells (CCL2 and CXCL8) contribute to inflammation during transplant rejection [108, 111]. Specifically, CCL3 is upregulated in allografts as early as 6 h after transplantation.

The early expression of CCL3 in liver allografts leads to increased intragraft inflammation by attracting recipient-derived NK cells [86] before T-cell infiltration [112]. It has been proven that graft-infiltrating NK cells are a major source of IFN-*γ*, which is an important immunoregulatory cytokine during early posttransplant period. The serum IFN-*γ* levels were markedly increased by day three after transplanation in recipients [86]. IFN-*γ*-producing NK cells are an important link between the innate and adaptive immune responses early after liver transplantation. So CC chemokines, NK cells, and innate immunity may be important in the early events leading to allograft rejection.

The C4d deposits along the portal capillaries in liver allografts indicate a humorally mediated immunoresponse caused by the accumulated B and plasma cells. During acute rejection, a significant increase of B lymphocytes and plasma cells, together with CCL20 (macrophage inflammatory protein-3alpha) and its receptor CCR-6, was detected in the portal fields of all biopsy specimens [87]. This result indicates that the migration of B lymphocytes and plasma cells promoted by the expression of B-cell activating chemokines/receptors plays a key role in acute liver rejection.

Chemokine receptors CCR2 and CCR5 are also found being upregulated on infiltrating lymphocytes and Kupffer cells during acute and chronic rejection [82]. Genes for the CC chemokine receptors CCR2 and CCR5 are characterized by polymorphisms which are associated with significant alterations in their function [113]. Fischereder study group reported that the homozygous expression of chemokine polymorphisms CCR5-delta 32 was associated with a significantly improved survival of renal transplant allografts [114]. However, genotyped DNA PCR or PCR-RFLP analysis in 207 liver transplant recipients suggested that the gene frequency of the CCR2-641, and CCR5-delta 32 alleles had no significant difference in recipients [115]. It was suggested that the CCR2-641 and CCR5-delta 32 polymorphisms did not influence the risk for acute rejection of liver allografts or graft survival [115, 116].

### 4.3. The CC Chemokines and Ischemic-Type Biliary Lesions

There is no doubt that the bile ducts are the Achilles' heel of the liver graft. Thus, ischemic-type biliary lesions (ITBL) are a life-threatening complication following liver transplantation [117], with an incidence varying between 5% and 26% [118, 119]. The prevalence of ITBL continues to increase with time after liver transplantation [120, 121]. ITBL is a radiological diagnosis, characterized by intra- and/or extrahepatic strictures and dilatations on a cholangiogram after orthotopic liver transplantation without any known cause. According to the features of ITBL (including bile duct stenoses, dilatations, and cast formation) and the therapeutic consequences, two major types can be distinguished: type A (the complete biliary system is affected) and type B (only the major extrahepatic bile ducts are involved). The pathological features of ITBL observed in liver allografts include the epithelial and muscular necrosis of the biliary system with periductal connective tissue being remarkably well preserved [27].

The underlying cause of ITBL remains unclear despite numerous studies [122]. Identified causes include ischemia-related injury, immunologically induced injury, and cytotoxic injury induced by bile salts. However, the etiology of ITBL appeared to be mostly related to ischemic injury [123]. Immunological injury is associated with ABO incompatibility, polymorphism in genes coding for chemokines, and pre-existing immunologically mediated diseases [118].

Chemokines CCL2 (monocyte chemotactic protein-1) is produced and secreted from cholangiocytes under pathological conditions [124]. It could result in the recruitment and activation of T cells, macrophages, and natural killer cells to protect against biliary infection [125]. CC chemokine receptor 5 (CCR5) and its ligands (CCL3 and CCL4) play a key role in postischemic and inflammatory damage [126]. CCR5 deta32 polymorphism is a mutant allele of CCR5 with an internal deletion of 32 base pair (bp). It has been shown to lead to a lower rate of acute rejection and improved survival after kidney transplantation [127]. For detecting CCR5-delta 32 polymorphism in blood samples of patients after liver transplantation, CCR5 polymerase chain reaction (PCR) analysis was performed in 146 recipients. Finally, 120 patients with wild-type CCR5 and 26 patients with CCR5-delta 32 were identified in this study. And ITBL occurred in 14 of 120 patients with wild-type CCR5 and in 8 of 26 patients with CCR5-delta 32 polymorphism. Compared to kidney transplantation, however, this study has suggested that CCR5-delta 32 is a significant risk factor for the development of ITBL after liver transplantation and leads to a reduction in grafts and recipient's survival [128].

## 5. The C Chemokine Family 

The C chemokine family includes XCL1 and XCL2 in humans, both of which bind the XCR1 receptor. XCL1, also named lymphotactin, activation-induced T-cell-derived and chemokine-related molecule (ATAC), and single C motif-1 (SCM-1), have just two cysteine residues, only one of which is located at the N-terminus. XCL2 (also called SCM-1*a*) is different from XCL1 by only two N-terminal amino acids. XCL1 is released from T cells, NK cells, NKT cells, **γ*/*δ** T cells, mast cells, and medullary thymic epithelial cells (mTECs) during infectious and inflammatory responses [14, 129], whereas XCR1 is expressed by a dendritic cell (DC) subpopulation (i.e., murine CD8+ DC and human CD141+ DC) [130] and is correlated with the ability of DCs to cross-present antigen [131].

XCL1 is essential for the medullary accumulation of thymic dendritic cell (tDCs) [132]. Naturally occurring regulatory T cells (nT reg cell) development is impaired in the thymus of X*cl1*-deficient mice; thymocytes generated in X*cl1*-deficient mice are potent in triggering and fail to establish self-tolerance [132]. Flow cytometry and PCR analysis showed a reduction in XCL1 and XCR1 expression, which was associated with the suboptimal regulatory function of Treg. Interestingly, Treg-mediated suppression and cytotoxicity in allergic asthma significantly increased when expression of XCL1 was upregulated [133]. So XCL1 and XCR1 are constitutively expressed in the thymus and regulate the thymic establishment of self-tolerance. Although XCL1 and XCR1 mRNA were not expressed in human liver tissue when analysed by the northern blot [62], disordered hepatocytes taken up by XCR1+ DCs will be digested and processed and hepatocellular antigens will be cross-presented by the MHC-class I molecules to CD8+ T cells [131]. Thus, the special ability of XCR1+ DCs contributes to self-tolerance in the innate and adaptive immune responses.

The XCL1-XCR1 axis plays an important role in DC-mediated cytotoxic immune response and critically contributes to the generation of thymic self-tolerance and development of regulatory T(Treg) cells [129]. Interestingly, considering restricted expression by human CD141+ DCs, XCR1 emerges as a prime candidate for vaccines designed to induce selective cytotoxic immunity.

## 6. The CX_3_C Chemokine Family 

The chemokine CX_3_CL1, also known as fractalkine, is the only member of the CX_3_C chemokine family. The two N-terminal cysteine molecules are separated by three different amino acids in CX_3_CL1 domain. CX_3_CL1, synthesized as a transmembrane protein with an extended mucin-like stalk on which a chemokine domain is located, is induced in activated primary endothelial cells. This CX3C chemokine can promote strong adhesion of T cells and monocytes [64].

CX_3_CR1 (C-X_3_-C motif receptor 1) is the only one known receptor corresponding to this chemokine. It is primarily expressed on circulating monocytes, tissue macrophages, tissue DC (dendritic cell), and T-cell and natural killer (NK) cell populations [134]. Recent studies have shown CX_3_CL1 in the pathogenesis of brain [135] and cardiac [136] disorders. However, CX_3_CL1mRNA was constitutively expressed in Kupffer cells and hepatocytes, especially the hepatocytes around the central veins [14]. And CX_3_CR1 expression has been identified on the biliary epithelium, hepatic stellate cells (HSCs), and hepatoma cell lines in the liver [137].

Serum concentrations of CX_3_CL1 and its specific receptor CX_3_CR1 were significantly elevated [138] in patients with chronic liver diseases at different stages of fibrosis progression. And a correlation was observed between serum CX_3_CL1 and quantitative serum fibrosis markers (i.e., hyaluronan or procollagen III peptide) in the patients [139]. However, real-time qPCR analysis showed a reduction in cx3cl1 and cx3cr1 intrahepatic expression in patients with different stages of liver fibrosis versus nonfibrotic livers [139]. Further studies have indicated that human HSCs downregulate CX_3_CR1 surface expression *ex vivo* [140] and that the number of activated HSCs may influence CX_3_CL1 serum levels [139].

CX_3_CR1 in the damaged liver promotes the survival of infiltrating monocytes and guides the differentiation of monocyte derived macrophages [44]. So more intrahepatic inflammatory cells and intrahepatic macrophage accumulation were observed in CX_3_CR1^−/−^ liver. And CX_3_CR1^−/−^ mice strikingly developed more progressive fibrosis than wild-type (WT) animals [139]. By activating anti-inflammatory signals in hepatic macrophages, the CX_3_CL1-CX_3_CR1 axis plays a protective function that limits liver inflammation and fibrosis *in vivo *[140]. These preliminary studies have thoroughly indicated that CX_3_CL1-CX_3_CR1 axis plays an important role in the pathogenesis and development of chronic liver allograft dysfunction. So pharmacological augmentation of fractalkine-CX_3_CR1 pathway may represent a potential therapeutic agent in liver diseases.

## 7. Experimentally Therapeutic Applications of Chemokines and Their Receptors 

The chemokines and their receptors axis represents a potential pharmacologic target for various human diseases. Especially, by recruiting and activating polymorphonuclear (PMN) cells and T cells into allografts during the progression of I/R injury, CXCL8 and its CXCR1/CXCR2 receptors contribute to the physiopathology of acute rejection [141] and increase the incidence of primary nonfunction, primary graft dysfunction, and biliary structures after liver transplantation. So present studies on therapeutic uses of chemokine receptor antagonists and blocking antibodies are emerging in the literatures. Generally, these recombinated/engineered small-molecule chemokine inhibitors can be divided into four types: antichemokine antibodies (mAb), chemokine antagonist, DNA plasmid encoding chemokine compounds, and chimerical chemokine compounds (or N-terminal modified chemokines) [142, 143]. Mainstream attention has been devoted to the development of ELR^+^ CXC chemokines receptor antagonists and blocking antibodies.

CXCR1 and CXCR2 are the two major chemokine receptors expressed on the surface of PMNs, endothelial cells and T cells in immune responses. Several classes of CXCR1 and CXCR2 antagonists have been reported in the literature. Reparixin (known as repertaxin) and DF 2156A are described as noncompetitive allosteric inhibitors of CXCL8 receptors CXCR1 and CXCR2 with optimal pharmacokinetic profiles. In rat model of liver I/R injury, they drastically inhibited PMN and monocyte/macrophage recruitment into reperfused livers and reduced liver damage in terms of alanine-aminotransferase levels and hepatocellular necrosis [141]. The experimental data, along with those showing a reduced reperfusion injury by anti-CXCL1, anti-CXCR2 antibodies, and CXCL8 receptor inhibitors [144, 145], clearly demonstrated the therapeutic potential of these ELR^+^ CXC chemokines receptors antagonists and blocking antibodies in the prevention of I/R injury and acute rejection in organ transplantation.

## 8. Summary

Despite the discoveries in chemokine biology that have led to important advances in our understanding of immune responses, angiogenesis, carcinogenesis, and cell cycle control over the last 25 years, the role of chemokines in chronic liver allograft dysfunction remains unanswered. The complex interactions between the chemokine superfamily and other cellular contributors shown in several studies are only just beginning to be mapped. Moreover, an understanding of the biological roles that chemokines play in the pathogenesis of chronic liver allograft dysfunction lags behind that for other conditions such as circulatory, respiratory, or haematological disorders. It is necessary for us to understand the chemokine superfamily and its functions in the organism from a perspective concerning chronic liver allograft dysfunction. Only by understanding the interaction of chemokines and their receptors will it be possible to design therapeutic strategies and pharmaceutical agents to ameliorate chronic liver allograft dysfunction and ultimately enhance long-term recipient and allograft survival after liver transplantation.

## Figures and Tables

**Table 1:** Causes of chronic liver allograft dysfunction.

Causes		Histopathologic changes and diagnosis
Immunological	Early allograft dysfunction	Early high transaminases persistent cholestasis and prolonged coagulopathy [4] acute hepatocellular damage or death.
	Acute rejection	Predominantly mononuclear portal inflammation containing neutrophils, lymphocytes, and eosinophils; inflammatory bile duct damage; and portal or central venous subendothelial inflammation or perivenular inflammation [22].
	Chronic rejection	A majority of small bile ducts damage bile duct loss affecting >50% of the portal tracts and foam cell obliterative arteriopathy [22].
	Chronic hepatitis	The presence of a portal and lobular mononuclear infiltrates in the absence of rejection or the graft damage caused by viral infection [3].
	De novo or recurrent autoimmune hepatitis	Significant titers (≥1 : 160) of smooth muscle antibodies and antinuclear antibodies interface hepatitis with portal lymphocytic infiltrates hypergammaglobulinemia and exclusion of viral infection or drug-induced hepatitis [23, 24].

Viral	De novo or recurrent viral hepatitis (HBV, HCV)	The portal inflammation tends to be more diffusely distributed throughout the portal tracts; lymphocytic cholangitis is limited to a minority of bile ducts [25].

Ischemia	Late effects of I/R injury	Hepatocytes and sinusoidal endothelial cells damage adhesion and aggregation of neutrophils and platelets in the sinusoids sinusoidal narrowing and elevated liver aminotransferase enzymes [26].
	Ischemic-type biliary lesions	The complete biliary system is affected or only the major extrahepatic bile ducts are involved. Epithelial and muscular necrosis of the biliary system and periductal connective tissue well preserved [27].

Toxic	Drugs and other toxins	Changes are usually mild and nonspecific like hepatitis, cholestasis, nodular regenerative hyperplasia, and veno-occlusive disease (sinusoidal congestion) or centrizonal necrosis [8].

Recurrent disease	Idiopathic posttransplantation hepatitis	Bile duct damage and venous endothelial inflammation chronic hepatitis that cannot be ascribed to a particular cause or presence of bridging fibrosis or cirrhosis [28].
	Recurrent PSC	Biliary strictures presence of mild portal edema mild nonspecific pericholangitis lamellar periductal edema concentric periductal fibrosis or biliary gestalt [29].
	Recurrent PBC	Noninfectious granulomatous cholangitis in the proper setting presence of antimitochondrial antibodies and absence of other causes such as infections and biliary strictures [30].
	Alcoholic and nonalcoholic steatohepatitis	Macrovesicular steatosis Mallory's hyaline ballooning cell degeneration perisinusoidal fibrosis and scattered neutrophilic inflammation [31, 32].

HBV: hepatitis B virus; HCV: hepatitis C virus; I/R injury: ischemia reperfusion injury; PSC: primary sclerosing cholangitis; PBC: primary biliary cirrhosis.

**Table 2:** Chemokines involved in the pathogenesis of liver disease.

Chemokine	Location in the liver	Function	Receptors and their distribution
*CXC *subfamily			
CXCL1	Hepatocytes, Kupffer cells, activated stellate cells; and endothelial cells [33, 34]	Neutrophil migration, innate immunity, acute inflammation, and angiogenesis	CXCR1: PMN, monocytes, and mast cells CXCR2: PMN, monocytes, and mast cells
CXCL2	Hepatocytes, Kupffer cells, activated stellate cells; and endothelial cells [34, 35]	Neutrophil migration, innate immunity, acute inflammation, and angiogenesis	CXCR2: PMN, monocytes, and mast cells
CXCL3	Hepatocytes, Kupffer cells, activated stellate cells, and endothelial cells [33, 34]	Neutrophil migration, innate immunity, acute inflammation, and angiogenesis	CXCR2: PMN, monocytes, and mast cells
CXCL4	Sinusoidal endothelium, platelets, NK cells, T cells, and neutrophils [36]	Chemotactic for neutrophils, monocytes, and fibroblasts	CXCR3: memory T cells, Th1, Th2, Th17, and Treg, NKT
CXCL4L1	Thrombin-stimulated platelets [37]	Angiogenesis and antitumoral chemokine	CXCR3: memory T cells, Th1, Th2, Th17, Treg, and NKT
CXCL5	Hepatocytes, Kupffer cells, and endothelial cells [33]	Neutrophil migration, innate immunity, acute inflammation, and angiogenesis	CXCR2: PMN, monocytes, and mast cells
CXCL6	Microvascular endothelial cells [38]	Neutrophil migration and innate immunity	CXCR1: PMN, monocytes, and mast cells CXCR2: PMN, monocytes, and mast cells
CXCL7	Platelets [39]	Angiogenesis, innate immunity, neutrophil migration, and regenerating vascular integrity after injury	CXCR1: PMN, monocytes, and mast cells CXCR2: PMN, monocytes, and mast cells
CXCL8	Endothelial cells, Kupffer cells, biliary epithelial cells, and stellate cells [34]	Innate immunity, neutrophil migration, and angiogenesis	CXCR1: PMN, monocytes, and mast cells CXCR2: PMN, monocytes, and mast cells
CXCL9	Sinusoidal epithelial cells [40]	Th1, Th2, Th17, and Treg recruitment and adaptive immunity	CXCR3: memory T cells, Th1, Th2, Th17, Treg, and NKT CCR3: eosinophils and basophils
CXCL10	Hepatocytes and liver sinusoids [15]	Th1, Th2, Th17, and Treg recruitment and adaptive immunity	CXCR3: memory T cells, Th1, Th2, Th17, Treg, and NKT CCR3: eosinophils and basophils
CXCL11	Liver sinusoids [15]	Th1, Th2, Th17, and Treg recruitment and adaptive immunity	CXCR3: memory T cells, Th1, Th2, Th17, Treg, and NK CCR3: eosinophils and basophils CCR5: monocytes, Th1 cells, and NK
CXCL12	Biliary epithelium [15]	Stem cell migration and B-cell lymphopoiesis	CXCR4: T and B cells, monocytes, stem cells, and NKT
CXCL13	Lymphocytes within microenvironments [41]	B-cell homing in lymphoid organ and liver and T-cell homing in the liver	CXCR5: B cells CXCR3: memory T cells, Th1, Th2, Th17, Treg and NKT
CXCL14	Macrophage and neutrophil [42]	Recruitment of monocytes/macrophages to the liver	Unknown
CXCL16	Liver sinusoids biliary epithelium [15]	T-cell migration, recruitment of CD4+ T cells, and CD8+ T cells and B cells into the liver	CXCR6: memory T cells, Th1, NK and NKT
CXCL17	Endothelial cells and hepatocellular carcinoma (HCC) cells [43]	Chemoattract DC and monocytes, angiogenesis, and carcinogenesis	Unknown

*CC *subfamily			
CCL1	Hepatocytes, hepatic stellate cells, endothelium, and Kupffer cells [44]	T-cell trafficking and Th2 response	CCR8: monocytes, Th2, Treg and NK
CCL2	Hepatocytes, Kupffer cells, and stellate cells [44]	Th1 inflammation, T-cell and monocyte migration, and innate and adaptive immunity	CCR2: monocytes, memory T cells, basophils and pDC
CCL3	Portal vessels, biliary epithelium, and sinusoidal endothelium [45, 46]	T-cell and monocyte migration, innate and adaptive immunity, inflammation,Th1 response, HIV infection, and hypersensitivity	CCR1: monocytes, memory T cells, Th1 and NK CCR5: monocytes, Th1 cells and NK
CCL4	Portal vessels, biliary epithelium, and sinusoidal endothelium [15]	Th1 response, adaptive immunity, inflammation, HIV infection	CCR5: monocytes, Th1 cells and NK
CCL5	Portal vessels, platelets, T-cells, macrophages, liver-derived dendritic cells, and Kupffer cells [15]	T cell and monocyte migration, innate and adaptive immunity, inflammation, Th1 response, and HIV infection, and hypersensitivity	CCR1: monocytes, memory T cells, Th1 and NK CCR3: eosinophils and basophils CCR5: monocytes, Th1 cells and NK
CCL7	Portal vessels, monocytes, endothelial cells, smooth muscle cells, and human CD34­ cells [47]	T-cell, NK cells, dendritic cells, basophils, eosinophils, and monocyte migration, innate and adaptive immunity, Th1 inflammation, and hypersensitivity	CCR1: monocytes, memory T cells, Th1, and NK CCR2: monocytes, memory T cells, basophils and pDC CCR3: eosinophils and basophils CCR5: monocytes, Th1 cells, and NK
CCL8	Portal and vascular endothelium [47]	T-cell and monocyte migration, innate and adaptive immunity, Th1 inflammation, hypersensitivity, and HIV infection	CCR1: monocytes, memory T cells, Th1, and NK CCR2: monocytes, memory T cells, basophils, and pDC CCR3: eosinophils and basophils CCR5: monocytes, Th1 cells, and NK
CCL11	Foetal liver [48]	Eosinophil and basophil migration, allergic inflammation, and Th2 response	CCR2: monocytes, memory T cells, basophils, and pDC CCR3: eosinophils and basophils CCR5: monocytes, Th1 cells, and NK CXCR3: memory T cells, Th1, Th2, Th17, Treg, and NKT
CCL12	Kupffer cells [49]	Monocytes, T-cell and eosinophils migration, and allergic inflammation	CCR2: monocytes, memory T cells, basophils, and DC
CCL13	Epithelial and endothelial cells [50]	T-cell and monocyte migration, innate and adaptive immunity, and Th1 inflammation	CCR1: monocytes, memory T cells, Th1, and NK CCR2: monocytes, memory T cells, basophils, and pDC CCR3: eosinophils and basophils
CCL14	Widely in liver and plasma [14]	T-cell and monocyte migration, hypersensitivity, innate and adaptive immunity, and inflammation	CCR1: monocytes, memory T cells, Th1, and NK CCR3: eosinophils and basophils CCR5: monocytes, Th1 cells, and NK
CCL15	Widely in liver [51]	T-cell, eosinophil, basophil, and monocyte migration, Th2 response, hypersensitivity, innate and adaptive immunity, and allergic inflammation	CCR1: monocytes, memory T cells, Th1, and NK CCR3: eosinophils and basophils
CCL16	Hepatocytes and biliary epithelial cells [52]	T-cell, eosinophil, basophil, and monocyte migration,Th2 response, hypersensitivity, innate and adaptive immunity, and allergic inflammation	CCR1: monocytes, memory T cells, Th1, and NK CCR3: eosinophils and basophils
CCL17	Keratinocytes, fibroblasts, stimulated monocytes, and certain DC [53]	T-cell and monocyte migration, allergic inflammation, and Treg retention	CCR4: Th2 cells, Treg eosinophils, basophils, DC, and Treg
CCL18	Portal area of livers with hepatitis C but not in normal livers [54]	Lymphocytes and immature DC activation	CCR3: eosinophils and basophils
CCL19	Portal-associated lymphoid tissue [15]	T-cell and DC homing to secondary lymphoid tissue and lymphoid development	CCR7: naive T, B, mature mDC, Th1, Th2, and Treg
CCL20	Endothelial cells, macrophages, and DC in the livers [55]	DC migration, memory T-cells, and Th17 cells at site of inflammation	CCR6: memory T cells, B cells, Th17, and immature mDC
CCL21	Portal-associated lymphoid tissue [15]	T-cell and DC homing to secondary lymphoid tissue; lymphoid development; T-cell recruitment; adaptive immunity; and Th1, Th2, Th17, and Treg inflammation	CXCR3 (mouse): memory T cells, Th1, Th2, Th17, Treg, and NKT CCR7: naive T, B, mature mDC, Th1, Th2, and Treg
CCL22	DC, B cell, and macrophages (Kupffer cells) [56]	T-cell and monocyte migration, allergic inflammation, Treg retention, and T-cell skin homing	CCR4: Th2 cells, Treg eosinophils, basophils, DC, and Treg
CCL23	Macrophages (Kupffer cells) [57]	Chemotactic activity on resting T cell, monocytes, and neutrophils	CCR1: monocytes, memory T cells, Th1, and NK
CCL24	Lower levels in the liver [58]	Eosinophil and basophil migration, allergic inflammation, and Th2 response	CCR3: eosinophils and basophils
CCL25	macrophages, Kupffer cells, DC, and cholangiocytes [59]	Recruitment of adaptive immune cells to the liver. T-cell homing to gut and thymus and tolerogenic DC	CCR9: DC, memory T cells, and thymocytes
CCL26	Vascular endothelial cells [60]	Eosinophil and basophil migration, allergic inflammation, and Th2 response	CCR3: eosinophils and basophils
CCL27	No expression in liver and predominantly in the skin keratinocytes [61]	T-cell trafficking in the skin	CCR10: memory T cells and Treg
CCL28	Biliary epithelium (cholangiocytes) [15]	T-cell homing to skin and bowel	CCR10: memory T cells and Treg

*C *subfamily			
XCL1	Not expressed in human liver [62]	NK-cell recruitment	XCR1: NK
XCL2	Preferentially in CD8+ T cells [63]	NK-cell recruitment	XCR1: NK

*CX3C *subfamily			
CX3CL1	Biliary epithelium [64]	Th1 inflammation, T-cell, NK cell trafficking and adhesion, and innate and adaptive immunity	CX3CR1: monocytes, and Th1, NK

CXCL: C-X-C motif chemokine ligand; CXCR: C-X-C motif chemokine receptor; CCL: C-C motif chemokine ligand; CCR: C-C motif chemokine receptor; XCL: X-C motif chemokine ligand; XCR: X-C motif chemokine receptor; CX3CL: C-X3-C motif chemokine ligand; CX3CR: C-X3-C motif chemokine receptor; Th1: T helper 1; Th2: T helper 2; Th17: T helper 17; Treg: regulatory T cell; NK: natural killer; NKT: natural killer T; DC: dendritic cell; pDC: plasmacytoid dendritic cell; mDC: myeloid dendritic cell; PMN: polymorphs.
